# Supplementary material for: Tumor Suppressors Having Oncogenic Functions: The Double Agents
Source: Cells. 2020 Dec 31;10(1):46. doi: 10.3390/cells10010046 (PMC7824251; doi:10.3390/cells10010046)
Supplement: Supplementary file 1 [file cells-10-00046-s001.pdf]

Supplementary Table S1: Mechanisms of mutant p53 GoF and their physiological effects

Mechanism		Interacting partners	Effects
Interaction with other TFs; regulation of transcription	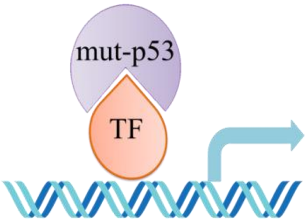	NF-Y [1,2], Sp-1 [3–5], PML [6]. C-Myc, Bcl-xl [7], ETS [8]	Altered gene expression and changes in cell phenotypes leading to proliferation and invasion
Interaction with family members p63 and p73; inhibition of transactivation	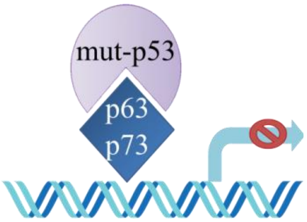	p63/p73 [9–12]	Decrease in growth inhibition leading to invasion, metastasis, migration, and chemoresistance
Formation of co-aggregates with wt-p53 and p63/ p73; inhibition of transactivation	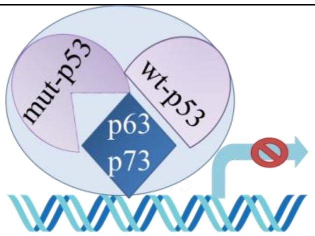	Co-tetramers with wt p53 [13] and/or super-tetramers with wt p53/p63/p73[14]	Abrogation of wild type activity, increased dominant negative effects, inhibited binding to target gene promoters and subsequent transactivation, NF-κβ pathway and Ras activation
Interaction with several proteins; alteration of functions	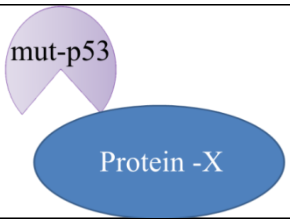	PML [15], Pin1[16], MRE11 [17], TOP1[18]	Disruption of DDR pathways, genomic instability, enhancing overall oncogenic activity of mut-p53
Direct recruitment to DNA, binding to sequence-specific DNA elements such as non-B DNA conformations or AT-rich regions; alteration of gene expression	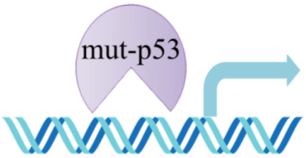	Putatively MYC, FOS, Bcl-Xl, PCNA, E2F, NFKB, TERT, VEGF, etc [19]	Enhanced proliferation, invasion and angiogenesis, and inhibition of apoptosis

Chromatin remodelling and chromatin accessibility	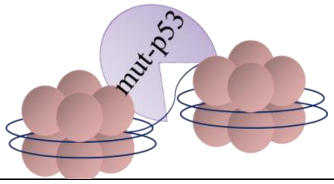	SWI/SNF [20], MLL1, MLL2, MOZ [21]	Enhanced expression of genes contained within specific regions of the chromatin
Cytoplasmic role	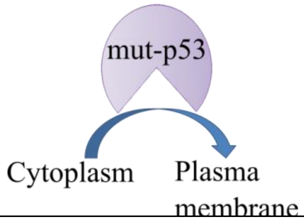	Shunting Glucose transporter GLUT1 to the plasma membrane [22]	Regulation of glucose uptake by cancer cells, supporting rapid proliferation (Warburg Effect)
Regulation of miRNA	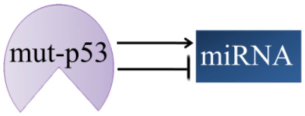	miR-16–1, miR-143, and miR-205 [23], miR-128-2 [24], miR-130b [25], miR-155 [26], miR-27a [27], DICER1 [28]	Transcriptional and post-transcriptional regulation of multiple miRNAs, suppression of apoptosis, increased EM and chemoresistance

TF: Transcription factor

References

1. Di Agostino, S.; Strano, S.; Emiliozzi, V.; Zerbini, V.; Mottolese, M.; Sacchi, A.; Blandino, G.; Piaggio, G. Gain of function of mutant p53: the mutant p53/NF-Y protein complex reveals an aberrant transcriptional mechanism of cell cycle regulation. *Cancer Cell* **2006**, *10*, 191–202, doi:10.1016/j.ccr.2006.08.013.
2. Liu, K.; Ling, S.; Lin, W.-C. TopBP1 mediates mutant p53 gain of function through NF-Y and p63/p73. *Mol. Cell. Biol.* **2011**, *31*, 4464–4481, doi:10.1128/MCB.05574-11.
3. Chicas, A.; Molina, P.; Bargonetti, J. Mutant p53 forms a complex with Sp1 on HIV-LTR DNA. *Biochem. Biophys. Res. Commun.* **2000**, *279*, 383–390, doi:10.1006/bbrc.2000.3965.
4. Bargonetti, J.; Chicas, A.; White, D.; Prives, C. p53 represses Sp1 DNA binding and HIV-LTR directed transcription. *Cell. Mol. Biol. Noisy--Gd. Fr.* **1997**, *43*, 935–949.
5. Torgeman, A.; Mor-Vaknin, N.; Zelin, E.; Ben-Aroya, Z.; Löchelt, M.; Flügel, R.M.; Aboud, M. Sp1-p53 heterocomplex mediates activation of HTLV-I long terminal repeat by 12-O-tetradecanoylphorbol-13-acetate that is antagonized by protein kinase C. *Virology* **2001**, *281*, 10–20, doi:10.1006/viro.2000.0779.
6. Kim, M.P.; Lozano, G. Mutant p53 partners in crime. *Cell Death Differ.* **2018**, *25*, 161–168, doi:10.1038/cdd.2017.185.
7. Huang, X.; Zhang, Y.; Tang, Y.; Butler, N.; Kim, J.; Guessous, F.; Schiff, D.; Mandell, J.; Abounader, R. A Novel PTEN/Mutant p53/c-Myc/Bcl-XL Axis Mediates Context-Dependent Oncogenic Effects of PTEN with Implications for Cancer Prognosis and Therapy. *Neoplasia N. Y. N* **2013**, *15*, 952–965.
8. Sampath, J.; Sun, D.; Kidd, V.J.; Grenet, J.; Gandhi, A.; Shapiro, L.H.; Wang, Q.; Zambetti, G.P.; Schuetz, J.D. Mutant p53 Cooperates with ETS and Selectively Up-regulates Human MDR1 Not MRP1. *J. Biol. Chem.* **2001**, *276*, 39359–39367, doi:10.1074/jbc.M103429200.

9. Strano, S.; Fontemaggi, G.; Costanzo, A.; Rizzo, M.G.; Monti, O.; Baccarini, A.; Del Sal, G.; Levrero, M.; Sacchi, A.; Oren, M.; et al. Physical interaction with human tumor-derived p53 mutants inhibits p63 activities. *J. Biol. Chem.* **2002**, *277*, 18817–18826, doi:10.1074/jbc.M201405200.
10. Di Como, C.J.; Gaiddon, C.; Prives, C. p73 function is inhibited by tumor-derived p53 mutants in mammalian cells. *Mol. Cell. Biol.* **1999**, *19*, 1438–1449, doi:10.1128/mcb.19.2.1438.
11. Gaiddon, C.; Lokshin, M.; Ahn, J.; Zhang, T.; Prives, C. A subset of tumor-derived mutant forms of p53 down-regulate p63 and p73 through a direct interaction with the p53 core domain. *Mol. Cell. Biol.* **2001**, *21*, 1874–1887, doi:10.1128/MCB.21.5.1874-1887.2001.
12. Li, Y.; Prives, C. Are interactions with p63 and p73 involved in mutant p53 gain of oncogenic function? *Oncogene* **2007**, *26*, 2220–2225, doi:10.1038/sj.onc.1210311.
13. Chan, W.M.; Siu, W.Y.; Lau, A.; Poon, R.Y.C. How Many Mutant p53 Molecules Are Needed To Inactivate a Tetramer? *Mol. Cell. Biol.* **2004**, *24*, 3536–3551, doi:10.1128/MCB.24.8.3536-3551.2004.
14. Xu, J.; Reumers, J.; Couceiro, J.R.; De Smet, F.; Gallardo, R.; Rudyak, S.; Cornelis, A.; Rozenski, J.; Zwolinska, A.; Marine, J.-C.; et al. Gain of function of mutant p53 by coaggregation with multiple tumor suppressors. *Nat. Chem. Biol.* **2011**, *7*, 285–295, doi:10.1038/nchembio.546.
15. Haupt, S.; di Agostino, S.; Mizrahi, I.; Alsheich-Bartok, O.; Voorhoeve, M.; Damalas, A.; Blandino, G.; Haupt, Y. Promyelocytic leukemia protein is required for gain of function by mutant p53. *Cancer Res.* **2009**, *69*, 4818–4826, doi:10.1158/0008-5472.CAN-08-4010.
16. Girardini, J.E.; Napoli, M.; Piazza, S.; Rustighi, A.; Marotta, C.; Radaelli, E.; Capaci, V.; Jordan, L.; Quinlan, P.; Thompson, A.; et al. A Pin1/mutant p53 axis promotes aggressiveness in breast cancer. *Cancer Cell* **2011**, *20*, 79–91, doi:10.1016/j.ccr.2011.06.004.
17. Song, H.; Hollstein, M.; Xu, Y. p53 gain-of-function cancer mutants induce genetic instability by inactivating ATM. *Nat. Cell Biol.* **2007**, *9*, 573–580, doi:10.1038/ncb1571.
18. Restle, A.; Färber, M.; Baumann, C.; Böhringer, M.; Scheidtmann, K.H.; Müller-Tidow, C.; Wiesmüller, L. Dissecting the role of p53 phosphorylation in homologous recombination provides new clues for gain-of-function mutants. *Nucleic Acids Res.* **2008**, *36*, 5362–5375, doi:10.1093/nar/gkn503.
19. Brosh, R.; Rotter, V. When mutants gain new powers: news from the mutant p53 field. *Nat. Rev. Cancer* **2009**, *9*, 701–713, doi:10.1038/nrc2693.
20. Pfister, N.T.; Fomin, V.; Regunath, K.; Zhou, J.Y.; Zhou, W.; Silwal-Pandit, L.; Freed-Pastor, W.A.; Laptenko, O.; Neo, S.P.; Bargonetti, J.; et al. Mutant p53 cooperates with the SWI/SNF chromatin remodeling complex to regulate VEGFR2 in breast cancer cells. *Genes Dev.* **2015**, *29*, 1298–1315, doi:10.1101/gad.263202.115.
21. Zhu, J.; Sammons, M.A.; Donahue, G.; Dou, Z.; Vedadi, M.; Getlik, M.; Barsyte-Lovejoy, D.; Al-awar, R.; Katona, B.W.; Shilatifard, A.; et al. Gain-of-function p53 mutants co-opt chromatin pathways to drive cancer growth. *Nature* **2015**, *525*, 206–211, doi:10.1038/nature15251.
22. Zhang, C.; Liu, J.; Liang, Y.; Wu, R.; Zhao, Y.; Hong, X.; Lin, M.; Yu, H.; Liu, L.; Levine, A.J.; et al. Tumour-associated mutant p53 drives the Warburg effect. *Nat. Commun.* **2013**, *4*, 2935, doi:10.1038/ncomms3935.
23. Suzuki, H.I.; Yamagata, K.; Sugimoto, K.; Iwamoto, T.; Kato, S.; Miyazono, K. Modulation of microRNA processing by p53. *Nature* **2009**, *460*, 529–533, doi:10.1038/nature08199.
24. Donzelli, S.; Fontemaggi, G.; Fazi, F.; Di Agostino, S.; Padula, F.; Biagioni, F.; Muti, P.; Strano, S.; Blandino, G. MicroRNA-128-2 targets the transcriptional repressor E2F5 enhancing mutant p53 gain of function. *Cell Death Differ.* **2012**, *19*, 1038–1048, doi:10.1038/cdd.2011.190.
25. Dong, P.; Karaayvaz, M.; Jia, N.; Kaneuchi, M.; Hamada, J.; Watari, H.; Sudo, S.; Ju, J.; Sakuragi, N. Mutant p53 gain-of-function induces epithelial-mesenchymal transition through modulation of the miR-130b-ZEB1 axis. *Oncogene* **2013**, *32*, 3286–3295, doi:10.1038/onc.2012.334.

26. Neilsen, P.M.; Noll, J.E.; Mattiske, S.; Bracken, C.P.; Gregory, P.A.; Schulz, R.B.; Lim, S.P.; Kumar, R.; Suetani, R.J.; Goodall, G.J.; et al. Mutant p53 drives invasion in breast tumors through up-regulation of miR-155. *Oncogene* **2013**, *32*, 2992–3000, doi:10.1038/onc.2012.305.
27. Wang, W.; Cheng, B.; Miao, L.; Mei, Y.; Wu, M. Mutant p53-R273H gains new function in sustained activation of EGFR signaling via suppressing miR-27a expression. *Cell Death Dis.* **2013**, *4*, e574, doi:10.1038/cddis.2013.97.
28. Su, X.; Chakravarti, D.; Cho, M.S.; Liu, L.; Gi, Y.J.; Lin, Y.-L.; Leung, M.L.; El-Naggar, A.; Creighton, C.J.; Suraokar, M.B.; et al. TAp63 suppresses metastasis through coordinate regulation of Dicer and miRNAs. *Nature* **2010**, *467*, 986–990, doi:10.1038/nature09459.
